# Cultural and Molecular Evidence of *Legionella* spp. Colonization in Dental Unit Waterlines: Which Is the Best Method for Risk Assessment?

**DOI:** 10.3390/ijerph13020211

**Published:** 2016-02-06

**Authors:** Savina Ditommaso, Monica Giacomuzzi, Elisa Ricciardi, Carla M. Zotti

**Affiliations:** Department of Public Health and Paediatrics, University of Turin, P.zza Polonia 94, Turin 10126, Italy; monica.giacomuzzi@unito.it (M.G.); elisa.ricciardi@unito.it (E.R.); carla.zotti@unito.it (C.M.Z.)

**Keywords:** *Legionella* spp., quantitative real-time PCR, enumeration, dentistry setting, total viable count

## Abstract

*Legionella* spp*.* are ubiquitous in aquatic habitats and water distribution systems, including dental unit waterlines (DUWLs). The aim of the present study was to determine the prevalence of *Legionella* in DUWLs and tap water samples using PMA-qPCR and standard culture methods. The total viable counts (TVCs) of aerobic heterotrophic bacteria in the samples were also determined. *Legionella* spp. were detected and quantified using the modified ISO 11731 culture method. Extracted genomic DNA was analysed using the iQ-Check Quanti *Legionella* spp. kit, and the TVCs were determined according to the ISO protocol 6222. *Legionella* spp. were detected in 100% of the samples using the PMA-qPCR method, whereas these bacteria were detected in only 7% of the samples using the culture method. The number of colony forming units (CFUs) of the TVCs in the DUWL and tap water samples differed, with the bacterial load being significantly lower in the tap water samples (*p*-value = 0). The counts obtained were within the Italian standard range established for potable water in only 5% of the DUWL water samples and in 77% of the tap water samples. Our results show that the level of *Legionella* spp. contamination determined using the culture method does not reflect the true scale of the problem, and consequently we recommend testing for the presence of aerobic heterotrophic bacteria based on the assumption that *Legionella* spp. are components of biofilms.

## 1. Introduction

*Legionella* spp. are ubiquitous in aquatic habitats and water distribution systems, including dental unit waterlines (DUWLs). Surveys have shown that the percentage of samples taken at different dental sites that were positive for *Legionella* spp. was highly variable and ranged from 0% to 100% [1,2,3,4,5,6]. The concentration of *Legionella* spp. in the water of dental units may reach 1000 organisms per mL [1]. The primary route of *Legionella* spp. transmission is inhalation or aspiration of environmentally contaminated aerosols [7,8,9].

The presumed natural reservoirs for this pathogen are amoebae that *Legionella* cells can invade and in which they can replicate. Amoebae are the natural hosts of legionellae in the environment; the relationship between these organisms is unique in that, amoebae which generally use other bacteria as food, are parasitized by legionellae [10]. Direct contact with or exposure to aerosols containing free-living amoebae may cause infections, and it has been suggested that amoebae can act a “Trojan horses” for pathogens such as *Legionella* [11,12]. Freshwater amoebae have been detected in dental unit water samples [13]. The intensive use of instruments such as air-water syringes, ultrasonic scalers, drills and bicarbonate-based air polishers in operative dentistry leads to the widespread creation of potentially pathogenic aerosols. Because the primary route of infection by *Legionella pneumophila* is inhalation, these aerosols represent a serious health problem for both patients and dental workers. Currently, despite this microbiological evidence and the millions of dental treatments provided over the years in industrialized countries, there is no published evidence of clusters or outbreaks of legionellosis linked to dental care. Notably, when isolates are not available for molecular typing, a connection to the source cannot be definitively confirmed. To date, only a 2012 case report of a healthy 82-year-old Italian woman who developed Legionnaires’ disease after a dental healthcare appointment [14] and a report of a fatal case of *Legionella*-based pneumonia in a dentist in the U.S. that was attributed to *L. dumoffi* [1] have been published. Considering that dental care workers experience daily cumulative exposure to aerosols created by dental waterlines and that there has been only one proven case of Legionnaires’ disease caused by exposure to dental waterlines, the occupational risk due to exposure to legionellae in DUWLs appears to be very low. According to Pankhurst [15], the infection risk category for immunocompetent healthcare workers ranges from 1 to 5 (low risk ≤ 8) for low levels of *Legionella pneumophila* in waterlines.

In addition, annual reports of legionellosis in various countries, including Italy [16], have described only a few cases yearly in which dental treatment has been reported as the only risk factor for the disease. The 2015 Italian guidelines for the prevention of Legionnaires’ disease [17] included for the first time safety recommendations for dental surgeries. All dentists are required to conduct a statutory risk assessment of their practices. To comply with their legal duties, employers must identify and assess the sources of risk and prepare a scheme for preventing and controlling risks. Moreover, they must monitor the quality of their DUWLs at least annually to ensure that the waterlines are “legionellae free”.

Cultivation is the principal approach to evaluating bacterial contamination employed in the past, but applying this approach to testing for *Legionella* spp. may result in false-negative data or underestimated bacterial counts. The rates of recovery of *Legionella* spp. using culture methods are generally markedly lower than 100% [18], due to the fastidious growth requirements of these bacteria, the overgrowth by other bacteria, and the legionellae damage/loss when samples are concentrated. In contrast, PCR methods can be used to detect non-culturable legionellae, those living within amoebae [19,20] and doublets or chains of *Legionella* cells, which are counted as only 1 CFU when using the culture method but can be counted as individual cells using qPCR.

In our previous study, both qPCR and propidium monoazide qPCR (PMA-qPCR) were used to test artificial samples and hot water system samples, and the results were compared with those obtained using traditional culture techniques [21]. The level of agreement between the results obtained using the two methods was 79%. Cohen’s kappa coefficient was calculated, which showed a moderate level of concordance between the results obtained using the two methods (κ = 0.586).

The aim of the present study was to determine the prevalence of *Legionella* in water from DUWLs and faucets in private dentistry settings using PMA-qPCR (a method for selectively quantifying viable *Legionella* cells) and standard culture methods (the commonly used method for environmental surveillance). We compared the suitability of these methods for the detection and enumeration of *Legionella* in a dentistry setting. We also determined the total viable counts (TVCs) of aerobic heterotrophic bacteria at 36 °C and 22 °C to evaluate the microbial quality of water obtained from the DUWLs and the relationship between the TVCs and *Legionella* counts.

## 2. Materials and Methods

From February to July 2015, 86 water samples were collected in 26 private dentistry settings. The dentistry offices were selected among those listed in the National Association of Italian Dentists (ANDI) of Torino (Italy). Water samples were collected from the DUWL and sink faucet (tap water) in each setting. The tap water sample was used as a control to verify the quality of the water supplied to the building in which the office was located. All of the tested DUWLs were directly connected to the municipal water supply, and 28 (47%) were equipped with disinfection systems; in addition, 16 of these DUWLs were continuously disinfected, and 12 were intermittently disinfected. The most commonly used chemical disinfection product (50%) was hydrogen peroxide.

### 2.1. Sampling Water from DUWLs

Water samples were collected in the morning from the air-water syringes and turbines of the DUWLs and were mixed together. Each sample was collected in a sterile 2-L plastic bottle containing sodium thiosulphate (10% *w*/*v*) and was divided into two equal parts for evaluation using culture and PMA-qPCR assays.

### 2.2. Sampling Tap Water

Before sampling, the taps were disinfected according to the following procedure: (1) the water-flow regulator was removed; (2) the interior of the tap was disinfected using a solution of sodium hypochlorite (10% *w*/*v*) for 2–3 min; (3) the entire faucet was disinfected using a Bunsen-burner flame and (4) the water was allowed to flow for 5 min. Subsequently, water samples were collected in a sterile 2-litre plastic bottle containing sodium thiosulphate (10% *w*/*v*).

### 2.3. Quantification of Legionella Using the Culture Method

The culture-based assays for detecting and quantifying *Legionella* were conducted according to the modified ISO 11731 method [22], which recommends the use of different media (the non-selective medium (BCYE) and the selective medium (MWY)) for routine water testing in hospitals. Using BCYE medium results in a high rate of positive samples and a much greater yield of *Legionella* spp. than does using MWY, whereas using the former medium is necessary to detect non-*L. pneumophila* spp., which grow poorly on selective media. Using MWY is necessary to recover *Legionella* spp. isolates, whereas the results of using BCYE are difficult to interpret due to the presence of contaminating background flora.

The water samples were concentrated 100-fold by filtration through a 0.2-μm pore polycarbonate filter (Millipore, Billerica, MA, USA). The filter membrane was aseptically placed in one of the bottom corners of a stomacher bag, and 10 mL of Page’s solution (pH 6.8) was added. The membrane was then rubbed using the finger and thumb of one hand for 1 min to detach the bacteria. Five millilitres of this concentrated sample was heat-treated in a water bath at 50° C for 30 min. The remainder of the concentrated sample was not treated. Aliquots of 0.2 mL of the untreated and heat-treated samples were spread on duplicate plates containing BCYE agar and MWY agar (Oxoid, Wesel, Germany). Under these experimental conditions, the limit of detection (LOD) was 50 CFU/L.

### 2.4. Quantification of Viable Legionella Using PMA-qPCR

The second aliquot of each sample was filtered through a 0.45-μm pore polycarbonate filter (Millipore, Billerica, MA, USA) according to the manufacturer’s instructions (Aquadien, Bio-Rad, Marnes-la-Coquette, France). The filter was overlaid with 500 μL of PMA (50 μM) in a 90-mm Petri dish and was then incubated in the dark for 10 min, after which it was placed on ice and exposed for 10 min to a 500-W light at a distance of 20 cm from the light source. After irradiation, the filter was placed in lysis solution for DNA extraction. To eliminate the bacterial resuspension step (which could cause the loss of some bacteria), the DNA was extracted directly from the bacteria on the filters. The conditions for this process were optimized in our previous study [23].

The extracted genomic DNA was analysed for the presence of amplifiable sequences using qPCR. qPCR analysis was performed using a iQ-Check Quanti *Legionella* spp. kit according to the manufacturer’s instructions (Bio-Rad, Marnes-la-Coquette, France). This kit is NF VALIDATION certified (certificate number BRD07/15-12/15) and contains the reagents needed to amplify and quantify a 100-bp fragment of the 5S rRNA gene of *Legionella* spp. This method allows for the quantification of *Legionella* in water samples in less than 3 h following the water sample filtration and DNA extraction steps. The limit of detection (LOD) of this qPCR method is 5 GU per well; performing the analysis using duplicate kits allowed us to achieve a total LOD of 80 GU/L. The limit of quantification (LOQ) was 10 GU**/**5 μL, corresponding to 608 GU/L. Each PCR reaction mixture contained an internal control (IC), which was a linear plasmid that should be amplified under all conditions. This control monitored the inhibitory effects that may have occurred in the reaction mixtures. The *Legionella* target and IC were always amplified in the same PCR well.

### 2.5. Quantification of Waterborne Bacteria

The effect of background bacteria on *Legionella* detection and enumeration in water samples from DUWLs was investigated by determining the total viable counts (TVCs) according to the ISO protocol 6222 [24]. One millilitre of undiluted samples and 1 mL of diluted samples (in sterile PBS) were tested using the pour-plate method on yeast extract agar. The number of colony-forming units (CFU) per mL of sample was calculated from the number of colonies that had formed on the medium after 7 days of incubation at 22 °C and after 5 days of incubation at 36 °C, according to the U.S. standard method [25].

### 2.6. Statistical Analysis

The qPCR data were analysed using Opticon Monitor Analysis Software version 3.4 (Bio-Rad, Hercules, CA, USA). The proportions were compared using Fisher’s exact test. The Mann-Whitney U test was adopted to evaluate the between-sample (tap water *vs.* DUWLs) differences in the viable cell counts of *Legionella* and in the TVC data. Correlation was evaluated using the Pearson test.

## 3. Results

### 3.1. Legionella Quantification Using Culture and PMA-qPCR Methods

During the analysis, inhibition of PCR amplification (of the IC and the gene target) was observed in only one DUWL water sample. After this sample was diluted, the reaction was positive for the internal control of the amplification kit and the target template. For all of the other samples, the established range of the kit (28 < cycle threshold < 42) was always satisfied.

The results obtained using the culture and PMA-qPCR methods to evaluate 86 water samples were compared. Twenty-six samples were taken from tap water, and 60 were obtained from DUWLs.

Overall, *Legionella* spp. were detected in 100% (86/86) of the samples using the PMA-qPCR method, whereas they were detected in only 7% (6/86) of the samples using the culture method. Of these six culture-positive samples, four were collected from the output water of DUWLs (6.6%), and two were collected from tap water (7.7%). According to Fisher’s exact test, the difference was not significant (Table 1).

**Table 1:** Prevalence of *Legionella* spp. in water samples, as determined using PMA-qPCR and culture method.

Type of Sample	Culture Method		PMA-qPCR	
	Positive n.	Negative n.	Positive n.	Negative n.
Tap water	2	24	26	0
DUWL output	4	56	60	0
Total No. (%)	6 (7%)	80 (93%)	86 (100%)	(0%)

(Fisher’s test = 1, *p* < 0.05).

Non-*pneumophila* *Legionella species* were isolated from four of the DUWL output water samples and one tap water sample, and *Legionella pneumophila* sg. 2–14 was isolated from one tap water sample. *Legionella* spp. were isolated from both the tap water and DUWL output in only one of the 26 clinics. Furthermore one clinic was positive for *Legionella* spp. in both of the DUWLs installed.

**Figure 1:**
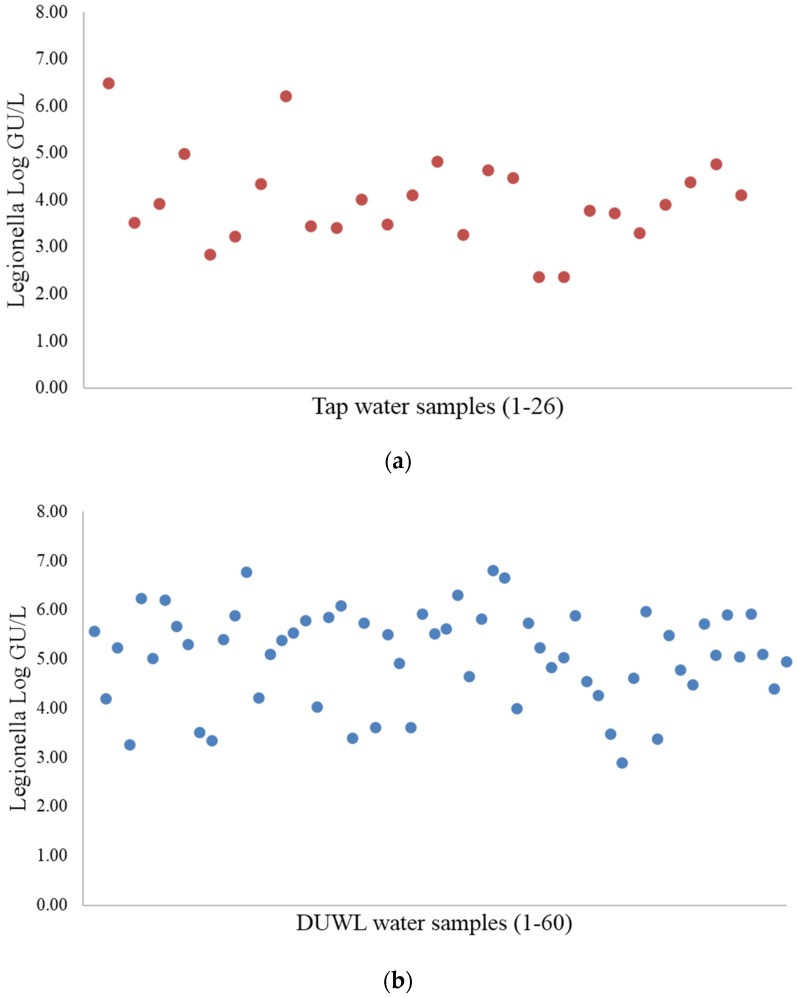
Quantitative results for (**a**) the tap water and (**b**) DUWL samples, as determined using the PMA-qPCR method.

The *Legionella* counts determined using PMA-qPCR ranged from 10^2^ to 10^6^ GU/L. The GU/L values for the 26 tap water samples and 60 DUWL samples determined using the PMA-qPCR method showed that the concentration of *Legionella* spp. was significantly lower (*p* = 0.000026) in the tap water samples, as determined the Mann-Whitney test (Figure 1).

Statistical consistency (determined using the Mann-Whitney test): U-value = 326; *p*-value = 0.000026. Of the six samples that tested positive using the culture method, five were found to contain less than 10^3^ CFU/mL of *Legionella*, and one contained >10^3^ CFU/mL (Table 2).

**Table 2:** Quantification of *Legionella* in the six culture-positive environmental water samples: comparison of the results obtained using the PMA-qPCR and culture methods and the TVCs.

Sample	*Legionella* PMA-qPCR	*Legionella* Culture	TVCs 22 °C	TVCs 37 °C
	(GU/L)	(CFU/L)	CFU/mL	CFU/mL
DUWL output water	6.7 × 10^4^	4.0 × 10^2^	3.0 × 10^3^	4.1 × 10^2^
DUWL output water	3.5 × 10^4^	1.0 × 10^2^	1.5 × 10^2^	2.6 × 10^2^
DUWL output water	1.1 × 10^5^	1.2 × 10^3^	1.4 × 10^3^	1.5 × 10^2^
DUWL output water	8.3 × 10^5^	9.0 × 10^2^	6.5 × 10^2^	70
Tap water	4.2 × 10^4^	8.0 × 10^2^	47	12
Tap water	5.2 × 10^3^	4.0 × 10^2^	5	1

### 3.2. Waterborne Bacteria

The number of colony forming units (CFUs) of the TVCs determined for the DUWL and tap water samples differed, with the bacterial load of the tap water samples being significantly lower (Table 3).

**Table 3:** Quantitative results obtained using TVCs.

Type of Sample	TVCs	
	22 °C	37 °C
Tap water (geometric mean CFU/L ± SD)	1.7 × 10^2^ ± 5.8 × 10^2^	1.3 × 10^3^ ± 5.8 × 10^2^
Dental unit (geometric mean CFU/L ± SD)	1.5 × 10^3^ ± 1.4 × 10^3^	1.3 × 10^3^ ± 2.0 × 10^3^

22 °C, U-value = 113.5; *p*-value = 0; 36 °C, U-value = 131; *p*-value = 0 (Mann-Whitney).

Comparing these results with the threshold values established by the European Council Directive 98/83/EC (20 CFU/mL at 36 °C and 100 CFU/mL at 22 °C) the counts were within the specified limits in only 5% (3/60) of the DUWL samples and in 77% (20/26) of the tap water samples.

*Legionella* spp. were isolated only from dental unit samples with bacterial loads ranging from 10^2^ CFU/mL to 3 × 10^3^ CFU/mL, whereas they were not isolated from any of the samples with a high bioburden (>3 × 10^3^ CFU/mL); in addition, two of the 20 tap water samples that satisfied the required standards for drinking water were contaminated with *Legionella* spp. (Table 2 and Table 4).

**Table 4:** Relationship between *Legionella-*positive cultures and the TVCs.

TVCs	DUWL Water Samples (n)	*Legionella*-Positive Cultures (n)	Tap Water Samples (n)	*Legionella*-Positive Cultures (n)
Drinking water threshold *	3	0	20	2
10^2^–3 × 10^3^ CFU/mL	31	4	5	0
>3 × 10^3^ CFU/mL	26	0	1	0
Total	60	4	26	2

* Threshold values established by the European Council Directive 98/83/E: <20 CFU/mL at 36 °C and <100 CFU/mL at 22 °C.

Overall, no significant association between the TVCs and the *Legionella*-positive culture results was demonstrated. We found that the TVC and PMA-qPCR detection methods had good accuracy, with 57 samples found to be *Legionella*-positive using both of these methods, yielding an accuracy rate of 95%.

## 4. Discussion

It has long been known that water samples collected at the outputs of dental unit waterlines are densely populated with microorganisms. For proliferation legionellae require the presence of other microorganisms (particularly amoebae), a supply of nutrients, and temperatures in the range of 20–45 °C [26]. As reported by many authors over the last 30 years, the rate of recovery of *Legionella* spp. from DUWLs ranges from 0% to 100%, and the counts of these bacteria in DUWL samples range from 0 to 10^6^ CFU/mL [1,27,28,29,30].

The very large variability in the counts reported in the literature is likely due to several factors, such as the amount of available soluble organic compounds, the presence of heavy metals, the temperature and level of free chlorine in municipal water distribution systems, the TVCs, the sampling time, and the analytical methods used (culture, PCR, or immunofluorescence staining) [31]. Due to its capacity to detect low levels of target nucleic acids, including those of viable *Legionella*, non-cultivable *Legionella* and *Legionella* within amoebae, q-PCR may be considered the gold standard method for determining the concentrations of these bacteria in water.

This study is the first to quantify *Legionella* in DUWLs using PMA-qPCR. In most previously published reports, the prevalence of *Legionella* in DUWLs has been evaluated using culture methods, and only Atlas [1], Williams [32] and Dutil [33] used PCR methods. However, in contrast with these previous studies, our chosen method eliminated the background of dead cells by including a PMA treatment, and despite employing this strategy, all of the samples tested positive for *Legionella*.

The rate of recovery of *Legionella* from the DUWLs observed in our study greatly varied depending on the analytical method used. When we used the culture method to detect *Legionella* in the DUWL samples, we observed a low rate of contamination of 6.6% (also observed in a previous study that has not yet been reported, in which we analysed 40 DUWL water samples using the culture method and found that none of the dental units tested had *Legionella-*contaminated water); however, when we used PMA-qPCR for the same purpose, we found that 100% of the samples were positive. These results, which were obtained using cold water samples (at 18–20 °C), are very different from those obtained using environmental samples collected from hospital water systems, most likely due to differences in their aqueous matrices. In our previous study [21], we analysed samples of hot water, an ideal habitat for *Legionella*, and the level of agreement between the results obtained using the two methods (culture *vs.* PMA-qPCR) was 79%. Consistent with the results of other studies [31,34,35], we demonstrated that although a significant proportion of the DUWLs tested were disinfected, samples taken from them were more heavily contaminated with bacteria compared with tap water samples collected from the same offices (by approximately 10-fold) and that their bacterial levels were much higher than the standards set for drinking water. Only three of the dental units studied delivered water that met the accepted Italian standard for drinking water (<20 CFU/mL at 36 °C and <100 CFU/mL at 22 °C). During sample collection, two of these DUWLs were treated with high concentrations of disinfectant. Our results confirm the findings of other studies [5,36], which have demonstrated that the concentration of heterotrophic bacteria is not associated with the number of *Legionella* isolates obtained using culture methods. However, we observed a good level of accuracy between the TVC and the PMA-qPCR method.

The three samples that were found to be *Legionella*-positive using the culture method were those with total bacterial counts ranging from 10^2^ to 10^3^ CFU/mL. The presence of other bacteria in biofilms, particularly that of Gram-negative bacteria that produce bacteriocins may inhibit the growth of *Legionella* or at least stress *Legionella* cells, making it impossible for *Legionella* to be detected using culture assays. Toze *et al.* [37] examined the ability of heterotrophic bacterial strains isolated from chlorinated drinking water on low-nutrient media to inhibit the growth of *Legionella* species. Sixteen to 32% of these strains inhibited the growth of *Legionella* spp. on buffered charcoal yeast extract agar, and the exact proportion of the growth-inhibiting strain varied according to the particular *Legionella* species. Moreover, when the amino acid supply becomes growth-limiting, intracellular bacteria produce factors that lyse the spent host cells, allowing the bacteria to survive osmotic stress, disperse in the environment and re-establish intracellular niches protected from lysosomal degradation. Arrival in a rich intracellular environment stimulates a return to the replicative phenotype. When the nutrient levels and other conditions of the host cells are favourable, *L. pneumophila* cells express factors that promote maximal replication [38,39].

In addition to inhibition due to other microorganisms, it must be remembered that the understimation by plate count method depends on the presence of *Legionella* bacteria contained in amoebae that produce and excrete vesicles. Rowbotham [14] has hypothesized that inhaling vesicles containing tightly packed legionellae is dangerous when these bacteria are motile. Greater than 90% of the observed vesicles fell within the size range considered to be respirable (1 to 5 µm in diameter) [11]. The *Legionella*-positivity observed using the qPCR method in the 3 samples compliant with drinking water standards could be linked to the presence of amoebae that are resistant to disinfection treatments.

## 5. Conclusions 

In summary, our results and the data obtained by Atlas [1] and Dutil [33] show high rates of *Legionella* contamination in DUWLs (from 68% to 93%), as determined by PCR analyses. Therefore, the presence of legionellae in dental units in which the effluent water is not treated with effective concentrations of chemical products, can be taken for granted because *Legionella* is an aquatic organism and the low temperatures of these units allow for its survival during starvation. Determining the *Legionella* concentrations in DUWL outputs using the culture method is not sufficient for predicting the exact size of the problem. We think that until the level of clinical risk is not expressed in GU/L, we cannot state that qPCR is the best method for the risk assessment. The concordance observed between the TVCs and PMA-qPCR results substantiates the assertion that the microbial load includes legionellae, regardless of their infectivity and pathogenicity. Therefore, we would like to share the suggestion of the CDC, which is that “… no rationale is seen for routine testing for such specific organisms” [40] and suggest that microbiological monitoring should not target individual organisms, such as *Legionella*, *Pseudomonas*, and *Acinetobacter* bacteria, but it should rather address the bioburden that encompasses them. Our final suggestion is that the cleanliness of dental units should be assessed by evaluating the concentrations of aerobic heterotrophic bacteria because contamination by these organisms are good indicators of the potential presence of *Legionella*.
